# A mixed‐methods investigation of a digital mental health tool to manage posttrauma anger

**DOI:** 10.1002/jts.23126

**Published:** 2025-01-26

**Authors:** Olivia Metcalf, Le Pham, Karen E. Lamb, Sophie Zaloumis, Meaghan L. O'Donnell, Tianchen Qian, Tracey Varker, Sean Cowlishaw, David Forbes

**Affiliations:** ^1^ Phoenix Australia–Centre for Posttraumatic Mental Health Department of Psychiatry University of Melbourne Carlton Victoria Australia; ^2^ Centre for Digital Transformation of Health University of Melbourne Parkville Australia; ^3^ Centre for Epidemiology and Biostatistics Melbourne School of Population and Global Health University of Melbourne Carlton Victoria Australia; ^4^ Methods and Implementation Support for Clinical Health Research Hub Faculty of Medicine Dentistry and Health Sciences University of Melbourne Parkville Victoria Australia; ^5^ Department of Statistics University of California Irvine Irvine California USA; ^6^ Turner Institute for Brain and Mental Health Monash School of Psychological Sciences Monash University Clayton Victoria Australia

## Abstract

Problematic anger affects up to 30% of individuals who have experienced trauma. Digital mental health approaches, such as ecological momentary assessment (EMA) delivered via smartphone and wearable devices (i.e., *wearables*), hold significant potential for the development of novel digital technology treatments. The objective of this cohort study was to examine the acceptability, feasibility, and outcomes from 10 days of usage of a digital mental health tool combining EMA and wearable use among trauma‐exposed adults with problematic anger. We used mixed methods to examine feasibility and acceptability and explored quantitative changes in mental health symptoms among participants over the study period (*N* = 98, 80.4% women, *M*_age_ = 38 years). Quantitative and qualitative data revealed that regular EMA combined with a wearable was feasible and acceptable in the sample. We observed reductions in problem anger, *p* < .001, repeated‐measures *d* (*d*_RM_) = ‐0.81, 95% CI [‐1.04, ‐0.59]; and posttraumatic stress disorder symptoms, *p* = .025, *d*_RM_ = ‐0.26, 95% CI [‐0.55, ‐0.03], over the 10 days of monitoring. Qualitative findings suggest that by regularly “checking in” on anger symptoms, participants improved their self‐awareness and ability to self‐manage their mood. These findings provide valuable learnings for building future personalized digital mental health tools.

Dysregulated anger, also termed *problem anger*, occurs independently in up to 30% of individuals who have experienced psychological trauma and can be evident in the hyperarousal symptoms characteristic of posttraumatic stress disorder (PTSD; Cowlishaw et al., 2021; Varker, Cowlishaw, et al., 2022). Problem anger is particularly challenging to treat using traditional psychological interventions, as individuals often present with high levels of hostility, mistrust, and low self‐awareness, which can impair the therapeutic alliance (Hyatt et al., 2023). Clinicians also report less confidence in managing clinical presentations marked by anger and hostility and a perception of being inadequately trained in this area, resulting in early treatment discharge. Furthermore, problem anger reduces the effectiveness of gold‐standard interventions for PTSD and poses a risk of relapse (Lloyd et al., 2014; Metcalf et al., 2023).

There is tremendous potential for smartphone and wearable‐delivered interventions in mental health, particularly in mental health concerns that are less responsive to standard approaches. Smartphones can deliver evidence‐based approaches for managing mental health, and sensors have the capability to track and alert the user to changes in their physiological state that may be linked to mood and behavior. Early work in this space has shown that ecological momentary assessment (EMA), a method of prompting users regularly to report their mood and behavior through microsurveys delivered via smartphone, may increase self‐awareness of mood states (Balaskas et al., 2021; Varker, Arjmand, et al., 2022). Wearable devices (i.e., *wearables*) are an additional digital health tool that can detect and alert users to physiological signs of stress. Such mechanisms are particularly pertinent to anger (Berkowitz & Harmon‐Jones, 2004), as, unlike internalizing disorders (e.g., depression, anxiety), anger is often an externally facing emotion (e.g., the individual scans the external environment for potential provocations or ruminates on how others and the world have wronged them). A lack of internal awareness of affective and physiological states reduces one's sensitivity to incremental increases in anger and irritability, contributing to the perception that a heightened expression of anger has come on suddenly and without warning. Thus, for anger, digital mental health tools that support increased self‐awareness may be of particular value and are in line with evidence‐based cognitive behavioral therapy (CBT) principles for anger management (Chemtob et al., 1997).

Very little research has been conducted using EMA and other digital mental health tools for problem anger. An EMA study of problem anger in male veterans found a reduction in problem anger symptoms after measurement (Varker, Arjmand, et al., 2022) and modeled associations between daily sleep quality and anger intensity (Arjmand et al., 2023). An earlier pilot randomized controlled trial by (Mackintosh et al., 2017) tested whether a digital mental health tool consisting of a mobile application (i.e., app) and wearable had added benefit compared to psychological treatment alone for problem anger in male veterans with PTSD, but the authors found no differences, which may reflect a ceiling effect of treatment efficacy. In that study, the wearable recorded heart rate data, and the app enabled users to record anger, access suggestions for behavioral strategies, and create daily personalized anger management plans. Problem anger can occur irrespective of a PTSD diagnosis, leaving room to explore these preliminary findings with a broader population. More recent work in a sample of clinically heterogeneous veterans with difficulties in anger, stress, and depression found that the use of a wearable to detect stress, in conjunction with standard CBT, reduced anger levels (Winslow et al., 2022).

The aim of the present study was to build an evidence base for novel digital mental health tools in the management of anger following trauma exposure. We had three specific research questions: (a) What is the feasibility and acceptability of using a smartphone and wearable digital tool in a trauma‐exposed population with problem anger?, (b) Are there observable changes in anger and PTSD symptoms among participants with problem anger who have undergone 10 days of EMA assessment combined with using a wearable?, and (c) What are the participant experiences of EMA and/or using the wearable that may account for any observed changes in clinical outcomes?

## METHOD

### Participants and procedure

#### Inclusion criteria, exclusion criteria, and screening

To be eligible for inclusion, participants had to be 18–50 years old; have problem anger, as indicated by a score of 12 or higher on the five‐item Dimensions of Anger Reactions Scale (DAR‐5; Forbes, Alkemade, Mitchell, et al., 2014); have experienced a previous traumatic event; and own their own internet‐connected smartphone. Participants were excluded from the study if they had been the perpetrator of severe physical violence (i.e., choking, assault with a weapon, assault that resulted in a serious injury) in the past 6 months, smoked regularly, and/or had conditions that affected the cardiovascular system. Participants were recruited from around Australia via social media, snowball sampling, and researchers’ databases. Participants were first screened online, then the research team screened potential participants a second time over the phone.

#### Study design

The method of this study is described in detail in the protocol.(Metcalf et al., 2022). In brief, this observational study followed a single group of participants, with data collected at three time points, defined as Time 1 (T1; predigital tool self‐report surveys), Time 2 (T2; use of a smartphone and wearable tool for a 10‐day period, occurring within 7 days of T1), and Time 3 (T3; postdigital tool self‐report surveys and qualitative interviews; see Table 1). Qualitative interviews were conducted with all participants over the Zoom app for an average duration of 55 min (range: 33–75 min; see Supplementary Material), during which participants were asked about their experiences with the smartphone app, wearable, and study protocol and asked to describe how the tool impacted them. Data were collected from August 2022 to March 2023. Using trauma‐informed research principles, participants were onboarded to the study via telephone, including downloading the study app, pairing and handling the wearable, and troubleshooting with an experienced researcher. Participants were reimbursed $250 (AUD) for their participation. The University of Melbourne Human Research Ethics Committee approved the study (Approval No. 2022‐22157‐28033‐5), and written informed consent was obtained electronically.

**TABLE 1:** Time points, constructs, and measures over the study period

Time point	Construct	Measure
Time 1	Demographic information	Gender, age, relationship, employment
Time 1	Trauma exposure	Life Events Checklist for *DSM‐5*
Time 3	Ease of use, Ease of understanding	On a scale of 1 to 10, how easy to use was the app? On a scale of 1 to 10, how easy to understand was the app? On a scale of 1 to 10, how easy to use was the wearable? On a scale of 1 to 10, how easy to understand was the wearable?
Time 1, Time 3	Problem anger	Dimensions of Anger Reactions Scale–5
Time 1, Time 3	State and trait anger	State–Trait Anger Expression Inventory–2
Time 1, Time 3	Posttraumatic stress disorder symptoms	Four‐item Posttraumatic Stress Disorder Checklist
Time 1, Time 3	Emotional self‐awareness	Emotional Self Awareness Scale
Time 1, Time 3	Anger rumination	Anger Rumination Scale
Time 2	Anger symptoms (four times per day)	In the past 3 hours, how much of the time did you feel angry or irritable? (0 = *never*, 1 = *little*, 2 = *somewhat*, 3 = *much*, 4 = *a great deal*) Rate the overall intensity of the anger or irritability you felt (1 = *lowest intensity*, 10 = *highest intensity*) When you felt angry or irritable, to what degree did you speak or shout about your anger or irritability? (0 = *never*, 1 = *little*, 2 = *somewhat*, 3 = *much*, 4 = *a great deal*) When you felt angry or irritable, to what degree did you do physical things like gesture, pound the table, slam doors, throw things, and so forth? (0 = *never*, 1 = *little*, 2 = *somewhat*, 3 = *much*, 4 = *a great deal*)
Time 2	Pain (four times per day), alcohol use (four times per day), and sleep quality (once per day)	How much pain are you currently experiencing? (Visual analog scale) In the past 3 hours, how many standard drinks did you consume? (0 = *no drinks*, 7 = *7+ drinks)* During the past night, how would you rate your sleep quality overall? (0 = *very good*, 1 = *fairly good*, 2 = *fairly bad*, 3 = *very bad*)

*Note*: *DSM‐5* = *Diagnostic and Statistical Manual of Mental Disorders* (5th ed.).

#### Digital tool

The digital tool consisted of four daily EMAs, which prompted the user to reflect on their anger frequency, intensity, and verbal and physical aggression, as well as sleep, alcohol use, and pain. EMAs were delivered via a customized study app (i.e., mEMA‐sense) in conjunction with a patient's own smartphone and a study‐loaned wearable (i.e., Garmin Vivofit 4). EMA items were drawn from previous studies (Varker, Arjmand, et al., 2022). The timing schedule was randomized within a personalized waking window, with the first EMA occurring within the first hour of waking and the remaining three EMAs occurring randomly within 4‐hr windows. Participants had 60 min to complete each EMA, with two reminders. The wearable alerted users when their physiological stress level (0–100, calculated as a converted heart rate variability [HRV] score) was high. No other intervention components in terms of behavior or mood management advice were provided, and participants did not have access to their EMA or wearable data.

### Measures

#### Lifetime trauma exposure

The Life Events Checklist for *DSM‐5* (LEC‐5; (Weathers, Blake, et al., 2013) was used to screen for lifetime exposure to potentially traumatic events. The LEC‐5 includes items related to 16 events that can result in PTSD, per Criterion A in the *Diagnostic and Statistical Manual of Mental Disorders* (5th ed; *DSM‐5*; American Psychiatric Association, 2013), along with an additional item querying exposure to any particularly stressful event not covered by the other items. Participants were asked to indicate their level of exposure for each item, with response options of “happened to me,” “witnessed it,” “learned about it,” “part of my job,” “not sure,” and “doesn't apply.”

#### Problem anger

Problem anger was measured using the DAR‐5 (Forbes, Alkemade, Mitchell, et al., 2014), a five‐item scale comprising four items that address anger responses and one item that addresses the impact of anger on the respondent's social relationships. The DAR‐5 has demonstrated strong internal validity in multiple studies, convergent validity with other measures of anger, and divergent validity with measures of depression (Forbes, Alkemade, Hopcraft, et al., 2014; Forbes, Alkemade, Mitchell, et al., 2014). Respondents are asked to indicate the frequency with which they experienced each item over the past 4 weeks, with responses rated on a Likert scale ranging from 1 (*none or almost none of the time*) to 5 (*all or almost all of the time*). A cutoff score of 12 or higher indicates the presence of problem anger. For T3, the reference point was adjusted to reflect past‐week frequency to avoid overlap with T1 data. In the current sample, the DAR‐5 demonstrated good internal reliability, Cronbach's α = .76.

#### Anger expression

Anger expression was measured using the State–Trait Anger Expression Inventory–2 (STAXI‐2; Spielberger, 1999), a 57‐item self‐report questionnaire developed to assess the experience, expression, and control of anger. The STAXI‐2 has demonstrated good internal reliability and concurrent validity (Spielberger, 1999) as well as good construct validity and reliability in clinical populations (Lievaart et al., 2016). In the current sample, the STAXI‐2 demonstrated good internal reliability, Cronbach's α = .84.

#### PTSD symptoms

An abbreviated, four‐item version of the original 20‐item PTSD Checklist for *DSM‐5* (PCL‐5; Weathers, Litz, et al., 2013) was used to measure PTSD symptoms (Price et al., 2016). This was due to the already lengthy nature of the T1 assessments and the fact that PTSD symptoms were not part of the inclusion criteria for the study, nor were they assessed during the study's EMA phase. The four‐item scale has previously demonstrated good specificity and comparable sensitivity relative to the full measure for screening for probable PTSD using a cutoff score of 10 (Price et al., 2016). The items included are *DSM‐5* Criterion B1 (intrusive thoughts), C2 (avoidance of external reminders), D2 (negative expectations), and E4 (easily startled). In the current sample, the abbreviated PCL‐5 demonstrated good internal reliability, Cronbach's α = .727.

#### Emotional self‐awareness

Emotional self‐awareness will be assessed with the Emotional Self‐Awareness Scale (ESAS; Kauer et al., 2012), a 33‐item, self‐report survey that measures emotional self‐awareness across five domains: recognition, identification, communication, contextualization, and decision‐making. In the initial study, the ESAS showed high internal consistency. Items are rated on a 5‐point Likert scale ranging from 0 (*never*) to 4 (*a lot*). Total scores range from 0 to 132, with higher scores indicating more emotional self‐awareness. In the current sample, the measure demonstrated good internal reliability, Cronbach's α = .76.

#### Anger rumination

The Anger Rumination Scale (ARS; Sukhodolsky et al., 2001) is a 19‐item scale that measures an individual's propensity to ruminate about events that have angered them. Participants were asked to indicate the degree to which they experienced particular ruminative thoughts (e.g., “I reenact the anger episode in my mind after it has happened,” “I think about the reasons people treat me badly,” and “I ruminate about my past anger experiences”), rating responses on a 4‐point Likert scale ranging from 1 (*almost never*) to 4 (*almost always*). Total scores are calculated by summing all scale items, with higher scores reflecting more anger rumination. The ARS has shown good internal consistency and test–retest reliability (Sukhodolsky et al., 2001). In the current sample, the ARS demonstrated good internal reliability, Cronbach's α = .93.

#### Ease of use and understanding

Ease of use and understanding was measured using quantitative self‐report measures of ease of use of the app and wearable (1 = *very difficult*, 5 = *very easy*), ease of understanding of the app and wearable (1 = *very difficult*, 5 = *very easy*), and levels of frustration with the study protocol (1 = *very frustrating*, 5 = *not at all*).

### Data analysis

To address the first research question, feasibility and acceptability were examined based on qualitative interview data as well as quantitative data related to how easy it was for participants to use and understand the app and wearable, how many participants responded to EMAs and used the wearable, the amount of missing EMA data from the 40 time points per participant, and the amount of missing stress‐related wearable data collected at the same time an EMA was delivered. Quantitative data were used to address the second research question, which was related to observable changes in anger and PTSD symptoms during the EMA period. To handle missing data in the analyses, summary statistics for the characteristics of participants who completed both the T1 and T3 assessments (complete responders) were compared with those for participants who only completed the T1 assessments (nonresponders) to assess whether there were any differences between these participants. Paired‐sample *t* tests were used to examine changes in the scores on the DAR‐5, STAXI‐2, abbreviated PCL‐5, ARS, and ESAS using T1 and T3 data. Repeated‐measures Cohen's effect size estimates (*d*_RM_), with 95% confidence intervals (CI), were produced using the *effsize* package in R (Version 4.0.3) to quantify the magnitude of participant within‐group change in each outcome. Data preparation was performed using SPSS (Version 25.0). No adjustments for multiple testing were undertaken.

To address the third research question (i.e., whether participant experiences of EMA and/or wearable use accounted for any observed changes in clinical outcomes), NVIVO (Version 14) was used to organize the data and assist in forming a coding tree. Two researchers performed thematic analysis using a six‐step “thematic analysis in psychology” approach (Braun & Clarke, 2006). Two researchers coded all of the data separately to ensure the reliability of the coding tree. Cohen's kappa coefficient was calculated and indicated good interrater reliability (i.e., greater than 0.75). Themes were then defined and revised through discussions between the two researchers. In reporting the findings, short excerpts (i.e. single words, short statements) and extended excerpts (i.e. longer statements) from the interview transcripts are presented as examples of themes. Some excerpts have been edited for brevity.

## RESULTS

### Demographic characteristics

Of the 508 individuals who completed the online screening, 137 were eligible for participation. The main reasons for ineligibility were age (*n* = 159), regular smoking or cardiovascular conditions (*n* = 106), not meeting the DAR‐5 cutoff score for problem anger (*n* = 62), use of severe physical violence in the past 6 months (*n* = 51), and no trauma exposure (*n* = 24); participants could meet more than one exclusionary criteria. In total, 98 participants enrolled in the study, 97 completed T1 data, 91 participants completed T2 (i.e., provided at least partial data for the 10 days of EMA and wearable period), and 89 individuals completed T3. Reasons for noncompletion included unexpected travel during the study window, rehospitalization for mental health concerns, change of mind, and loss to follow‐up. Participants were primarily women (80.4%) and ranged in age from 18 to 60 years old (*M* = 37.8 years, *SD* = 10.0). At T1, 81.6% of participants had probable PTSD, 61.9% had moderate‐to‐severe levels of pain, and 92.8% had moderate‐to‐severe insomnia. Participants had experienced an average of just over four traumatic life events (*M* = 4.31, *SD* = 2.54); 3.1% of the sample reported military trauma, 96.9% reported civil traumatic events, and 43.3% of participants had suffered at least one sexual assault. Approximately 37% of participants were receiving some treatment for their mental health, the majority of these in the form of taking antidepressant medication and/or engaging with a primary care clinician; no participants were receiving anger‐focused treatment. Logistic regressions were conducted to examine the differences between nonresponders at T3 (i.e., missing; *n* = 8) and complete responders regarding sociodemographic characteristics including gender, age group, educational attainment, and marital status, as well as psychometric variables. These logistic regressions showed no statistically significant impacts of missingness, which enabled the management of missingness through pairwise deletion.

### Feasibility and acceptability

T3 data from the 89 completers revealed that the study app was rated highly regarding ease of understanding (*M* = 4.45, *SD* = 0.68) and use (*M* = 4.31, *SD* = 0.71); the wearable was also rated highly for both ease of understanding and use (*M* = 4.18, *SD* = 0.83 and *M* = 4.34, *SD* = 0.82, respectively). Participants reported that overall, frustration with downloading and connecting the app and wearable was low (*M* = 1.73), and privacy concerns were low (*M* = 1.81)​. Nearly the entire sample (89.9%) reported that 10 days was an acceptable period, and only 3.5% of the sample said they would not participate in a 30‐day digital mental health study.

Missingness indicated that over the 10 days of T2, a total of 3,599 EMA surveys were received by 91 participants, with 2,611 EMA surveys completed (72.5%) and 91 participants completing at least one EMA. Three participants had no data from the wearable. Furthermore, 34.9% of observations (*n* = 1,257) from 88 participants had both EMA and stress‐related wearable data available at the time of a given EMA prompt. Qualitative data revealed that all participants reported charging and wearing the wearable. The main reasons for missing EMA data were that the individual was driving, was at work, or experienced technical issues with the app.

### Psychometric measures

Only eight (8.2%) participants did not provide data for both T1 and T3. Paired scores of the 89 participants for the DAR‐5, ARS, ESAS, abbreviated PCL‐5, and STAXI‐2 at T1 and T3 are shown in Table 2, which includes descriptive statistics (i.e., means and standard deviations) for both time points as well repeated‐measures effect size estimates (*d*_RM_). Statistically, reductions were significant for anger symptoms (i.e., DAR‐5 and STAXI‐2), anger rumination, and PTSD symptoms. For the DAR‐5, the *d*_RM_ estimate corresponded to a large improvement over time, *d*_RM_ = ‐0.81, whereas the *d*_RM_ estimates indicated small‐to‐medium improvements for anger rumination and PTSD symptoms (Table 2).

**TABLE 2:** Changes in psychological measures among participants with problem anger before and after using the digital tool

	Time 1		Time 3				Effect size	
Variable	*M*	*SD*	*M*	*SD*	*t*(88)	*p*	*d*_RM_	95% CI
DAR‐5	15.94	3.41	13.08	3.62	−8.331	< .001	−0.81	[−1.04, ‐0.59]
ARS	51.19	11.04	49.11	11.14	−2.548	.013	−0.19	[−0.48, 0.11]
ESAS	69.81	11.18	68.04	10.85	−1.809	.074	−0.16	[−0.46, 0.14]
PCL‐5	9.60	3.24	8.93	3.43	−2.274	.025	−0.26	[−0.55, 0.03]
STAXI	52.94	10.09	50.82	11.17	2.826	.060	0.20	[−0.50, 0.10]

*Note*: *n* = 89. RM = repeated measures; CI = confidence interval; DAR‐5 = Dimensions of Anger Reactions Scale; ARS = Anger Rumination Scale; ESAS = Emotional Self‐Awareness Scale; PCL‐5 = PTSD Checklist for *DSM‐*5; STAXI = State–Trait Anger Expression Inventory–2.

### Digital health tool experiences

Qualitative results revealed that the app and wearable offered positive experiences for participants, with five key themes identified: promoting healthy behaviors, increasing self‐awareness, supporting self‐management, making the mind–body connection, and the potential to support other treatments.

#### Promoting healthy behaviors

Many participants perceived that the tool was very useful as a journal, diary, or health reminder that aided them in keeping track of their daily mental health as it pertained to things such as sleep and their anger. One participant noted, “Having the surveys throughout the day was a helpful reminder to check in with myself and just notice the, maybe, shifting levels of frustration or anger across the day and just really tune into that.” Other participants noted that the tool kept them more conscious of unhealthy coping behaviors, like drinking. One participant said, “I noticed when I was doing the alcohol questions, I actually do become more irritable when I'm drinking.”

#### Increasing self‐awareness

Participants reported that the tool made them more self‐aware about their anger and triggers. One participant stated:

It kind of was a bit of an eye‐opener…it made me more mindful going forward. I found it [to be like] self‐help—made you think about what anger is and what counts as anger and whether I had really been angry and those triggers.

In addition, other participants reported the tool benefited their self‐awareness of a wide range of emotions, with one noting:

The app made me much more self‐aware of my emotions…especially during the times when I had to think about what emotion I was feeling at the time. I normally don't think about that until I'm coming out of that distressed state.

Finally, some participants found that the increase in self‐awareness was comforting in that they realized their levels of anger were better than they thought: “When I look back on it from my side, I think I only got quick‐tempered about three times…I think I'm more placid than I thought.”

#### Supporting self‐management

Participants reported positive experiences with the mood‐monitoring approach of regular check‐ins for managing both mood and behavior related to anger. As one participant noted:

When I was asked to do a check‐in, it kind of gave me a moment to step out of the…not physically step out but take myself out of the situation. If I was getting angry, it just pulled me out and allowed me to be more aware of what was happening around me and make better decisions.

#### Making the mind–body connection

Several participants noted that the wearable alerted them to their physiological signs of anger, which, in turn, helped them self‐manage their anger symptoms. One participant stated:

What the watch did help me to do was look at my heart rate because I don't wear a smartwatch, so especially on the hot days in the city or when I was frustrated, I'd look at the watch to look at my heart rate, and I'd try to bring it down by breathing or maybe relaxing or sitting down and focus[ing] on my body.

#### Potential to support treatment

Some participants reported finding the tool useful for the current treatment of their mental health problems and associated symptoms, with one participant stating:

I like that it gave me more things to talk about with my counselor…potentially some more things to discuss with them that might have to result in us working out different ways to add something like this into my day because I did find it helpful.

#### Negative experiences

Participants were also probed about negative experiences. Overall, 67.3% of the sample reported nothing negative. The most common negative experiences were that EMA questions were not personalized enough to participants’ own experiences (10.4%), EMA questions were repetitive (8.1%), there were technical issues with the app or wearable (4.7%), and participants would prefer to use their own wearable (4.7%). Only one participant found that reflecting on their aggression was distressing, but they reported they would still participate in future studies of the same nature.

## DISCUSSION

The aim of this study was to investigate the potential for smartphone and wearable tools for individuals who have problem anger and have experienced trauma, both understudied populations in the digital mental health field. The goal of this study was not to conclusively establish that digital mental health tools are clinically effective but rather to grow an evidence base supporting the future development of EMA‐based interventions. The data were collected primarily from women, possibly due to exclusion criteria related to risk, which prevented individuals who had used severe violence in the past 6 months from participating. Most participants had experienced multiple traumatic events, averaging four per person, and presented with co‐occurring PTSD symptoms, problem anger, and high levels of sleep disturbances and pain. These findings expand upon earlier research among male veterans with PTSD (Morland et al., 2016) as well as a clinically heterogeneous group of veterans with anger (Winslow et al., 2022), showing that smartphone‐based and wearable tools have significant potential for addressing problem anger following trauma exposure.

The value of the smartphone app and wearable was two‐fold in that the study procedure was highly acceptable to the population and there were observed—albeit uncontrolled—improvements in anger symptoms as assessed using the DAR‐5 and STAXI‐2, along with improvements in anger rumination and PTSD symptoms after 10 days. The causes of these changes remain unclear. The reported symptom improvements are consistent with a previous study using EMA to measure problem anger in male veterans (Varker, Arjmand, et al., 2022). The tool itself had minimal intervention components: The primary focus of the combined smartphone app and wearable was to help in the self‐monitoring of anger, with the wearable detecting and alerting users to high levels of physiological stress. The observed symptom changes may be the result of measurement artifacts, including individual changes in perceptions of anger over time, and the differences in reference points used for the DAR‐5. Alternatively, regression toward the mean may explain these results, further highlighting the need for controlled EMA studies. Equally, unobserved variables may explain the change, and the lack of certainty around this finding reflects the dearth of literature on posttrauma problem anger more broadly.

Qualitative data indicated that users felt the tool improved their self‐awareness and self‐management of their anger and that the wearable helped them to make the mind–body link between physiological arousal and mood states. Interestingly, although no instructions were delivered, the qualitative data indicates that EMA approaches may result in the spontaneous self‐management of mood and behavior. Further research is needed, including EMA studies with a control group, to determine whether mood and physiological monitoring approaches in problem anger may be effective via potential mechanisms of increasing internal focus. Though this holds promise for the development of digital mental health tools that incorporate EMA, it raises questions regarding the validity of EMA as a pure measurement approach in anger. Furthermore, there were no changes in emotional self‐awareness. This may reflect the fact that the tool focused specifically on anger awareness rather than global emotional awareness, whereas previous studies have found that nonspecific mood monitoring improves emotional self‐awareness (Kauer et al., 2012).

The goal of this study was to expand a new area of research. There are many limitations of this study. Importantly, there was no control group. Designing active control groups for EMA studies is challenging, and much remains unknown about EMA research in anger. Of note, previous research shows that recalling anger differs between longer and shorter periods of time, and changes in anger symptoms observed in this study may be influenced by changes in the accuracy of one's perceptions (Winkielman et al., 1998). In addition, the reference points for the DAR‐5 were adjusted, which also likely impacted the results. For safety reasons, the sample was also restricted to exclude individuals who reported engaging in significant physical violence, resulting in a sample with majority women. Importantly, this population remains significantly understudied relative to men with problem anger, but how these findings relate to male populations remains unknown. Finally, to reduce participant burden, we used a shorter measure of PTSD symptoms, which may not accurately reflect symptom changes. These exploratory findings support further research into more sophisticated digital mental health tools for problem anger, such as ecological momentary interventions, “just‐in‐time” adaptive interventions, and novel digital tools that leverage wearables.

## OPEN PRACTICES STATEMENT

The study reported in this article was not formally preregistered. Neither the data nor the materials have been made available on a permanent third‐party archive; requests for the data or materials should be sent via email to the lead author at Olivia.Metcalf@unimelb.edu.au.

## AUTHOR NOTE

Funding was provided by the National Health and Medical Research Council.

## Supporting information



Supporting Material

## References

[jts23126-bib-0001] American Psychiatric Association . (2013). Diagnostic and statistical manual of mental disorders (5th ed.). 10.1176/appi.books.9780890425596

[jts23126-bib-0002] Arjmand, H. ‐A. , Forbes, D. , Varker, T. , O'Donnell, M. L. , Finlayson‐Short, L. , & Metcalf, O. (2023). Understanding the temporal dynamics of problem anger using sequence analysis. Emotion, 23(8), 2322–2330. 10.1037/emo0001240 37053411

[jts23126-bib-0003] Balaskas, A. , Schueller, S. M. , Cox, A. L. , & Doherty, G. (2021). Ecological momentary interventions for mental health: A scoping review. PloS One, 16(3), Article e0248152. 10.1371/journal.pone.0248152 PMC7951936 33705457

[jts23126-bib-0004] Berkowitz, L. , & Harmon‐Jones, E. (2004). Toward an understanding of the determinants of anger. Emotion, 4(2), 107–130. 10.1037/1528-3542.4.2.107 15222847

[jts23126-bib-0005] Braun, V. , & Clarke, V. (2006). Using thematic analysis in psychology. Qualitative Research in Psychology, 3(2), 77–101. 10.1191/1478088706qp063oa

[jts23126-bib-0006] Chemtob, C. M. , Novaco, R. W. , Hamada, R. S. , & Gross, D. M. (1997). Cognitive–behavioral treatment for severe anger in posttraumatic stress disorder. Journal of Consulting and Clinical Psychology, 65(1), 184–189. 10.1037//0022-006x.65.1.184 9103748

[jts23126-bib-0007] Cowlishaw, S. , Metcalf, O. , Varker, T. , Stone, C. , Molyneaux, R. , Gibbs, L. , Block, K. , Harms, L. , MacDougall, C. , & Gallagher, H. C. (2021). Anger dimensions and mental health following a disaster: Distribution and implications after a major bushfire. Journal of Traumatic Stress, 34(1), 46–55. 10.1002/jts.22616 33136348

[jts23126-bib-0008] Forbes, D. , Alkemade, N. , Hopcraft, D. , Hawthorne, G. , O'Halloran, P. , Elhai, J. D. , McHugh, T. , Bates, G. , Novaco, R. W. , & Bryant, R. (2014). Evaluation of the Dimensions of Anger Reactions–5 (DAR‐5) Scale in combat veterans with posttraumatic stress disorder. Journal of Anxiety Disorders, 28(8), 830–835. 10.1016/j.janxdis.2014.09.015 25445072

[jts23126-bib-0009] Forbes, D. , Alkemade, N. , Mitchell, D. , Elhai, J. D. , McHugh, T. , Bates, G. , Novaco, R. W. , Bryant, R. , & Lewis, V. (2014). Utility of the Dimensions of Anger Reactions–5 (DAR‐5) scale as a brief anger measure. Depression and Anxiety, 31(2), 166–173. 10.1002/da.22148 23801571

[jts23126-bib-0010] Hyatt, C. S. , Sleep, C. E. , Hemmy Asamsama, O. , & Reger, M. A. (2023). Surveying Veterans Affairs mental health care providers on experiences working with veteran patients with antagonistic clinical presentations. Psychological Services, 21(2), 379–387. 10.1037/ser0000782 37358554 PMC10749978

[jts23126-bib-0011] Kauer, S. D. , Reid, S. C. , Crooke, A. H. D. , Khor, A. , Hearps, S. J. C. , Jorm, A. F. , Sanci, L. , & Patton, G. (2012). Self‐monitoring using mobile phones in the early stages of adolescent depression: Randomized controlled trial. Journal of Medical Internet Research, 14(3), Article e1858. 10.2196/jmir.1858 PMC3414872 22732135

[jts23126-bib-0012] Lievaart, M. , Franken, I. H. , & Hovens, J. E. (2016). Anger assessment in clinical and nonclinical populations: Further validation of the State–Trait Anger Expression Inventory–2. Journal of Clinical Psychology, 72(3), 263–278. 10.1002/jclp.22253 26766132

[jts23126-bib-0013] Lloyd, D. , Nixon, R. , Varker, T. , Elliott, P. , Perry, D. , Bryant, R. , Creamer, M. , & Forbes, D. (2014). Comorbidity in the prediction of cognitive processing therapy treatment outcomes for combat‐related posttraumatic stress disorder. Journal of Anxiety Disorders, 28(2), 237–240. 10.1016/j.janxdis.2013.12.002 24507630

[jts23126-bib-0014] Mackintosh, M. ‐A. , Niehaus, J. , Taft, C. T. , Marx, B. P. , Grubbs, K. , & Morland, L. A. (2017). Using a mobile application in the treatment of dysregulated anger among veterans. Military medicine, 182(11–12), e1941–e1949. 10.7205/MILMED-D-17-00063 29087863

[jts23126-bib-0015] Metcalf, O. , Finlayson‐Short, L. , Forbes, D. , O'Donnell, M. , & Varker, T. (2023). A systematic review of treatments for problem anger in veteran and military populations with PTSD. Aggression and Violent Behavior, 70, 1–9. 10.1016/j.avb.2023.101837

[jts23126-bib-0016] Metcalf, O. , Finlayson‐Short, L. , Lamb, K. E. , Zaloumis, S. , O'Donnell, M. L. , Qian, T. , Varker, T. , Cowlishaw, S. , Brotman, M. , & Forbes, D. (2022). Ambulatory assessment to predict problem anger in trauma‐affected adults: Study protocol. PLoS One, 17(12), Article e0278926. 10.1371/journal.pone.0278926 PMC9778625 36548307

[jts23126-bib-0017] Morland, L. A. , Niehaus, J. , Taft, C. , Marx, B. P. , Menez, U. , & Mackintosh, M.‐A. (2016). Using a mobile application in the management of anger problems among veterans: A pilot study. Military Medicine, 181(9), 990–995. 10.7205/MILMED-D-15-00293 27612342

[jts23126-bib-0018] Price, M. , Szafranski, D. D. , van Stolk‐Cooke, K. , & Gros, D. F. (2016). Investigation of abbreviated 4‐ and 8‐item versions of the PTSD Checklist–5. Psychiatry Research, 239, 124–130. 10.1016/j.psychres.2016.03.014 27137973

[jts23126-bib-0019] Spielberger, C. D. (1999). STAXI‐2: State–Trait Anger Expression Inventory‐2: Professional manual. Psychological Assessment Resources.

[jts23126-bib-0020] Sukhodolsky, D. G. , Golub, A. , & Cromwell, E. N. (2001). Development and validation of the Anger Rumination Scale. Personality and Individual Differences, 31(5), 689–700. 10.1016/S0191-8869(00)00171-9

[jts23126-bib-0021] Varker, T. , Arjmand, H. , Metcalf, O. , Cowlishaw, S. , O'Donnell, M. , Forbes, D. , McFarlane, A. , Bryant, R. A. , Hopwood, M. , Phelps, A. , & Hinton, M. (2022). Using an ecological momentary assessment protocol to understand problem anger in veterans. Journal of Behavior Therapy and Experimental Psychiatry, 76, Article 101746. 10.1016/j.jbtep.2022.101746 35738692

[jts23126-bib-0022] Varker, T. , Cowlishaw, S. , Baur, J. , McFarlane, A. C. , Lawrence‐Wood, E. , Metcalf, O. , Van Hooff, M. , Sadler, N. , O'Donnell, M. L. , & Hodson, S. (2022). Problem anger in veterans and military personnel: Prevalence, predictors, and associated harms of suicide and violence. Journal of Psychiatric Research, 151, 57–64. 10.1016/j.jpsychires.2022.04.004 35453092

[jts23126-bib-0023] Weathers, F. W. , Blake, D. D. , Schnurr, P. P. , Kaloupek, D. G. , Marx, B. P. , & Keane, T. M. (2013). The Life Events Checklist For DSM‐5 (LEC‐5) . https://www.ptsd.va.gov/professional/assessment/te‐measures/life_events_checklist.asp

[jts23126-bib-0024] Weathers, F. W. , Litz, B. T. , Keane, T. M. , Palmier, P. A. , Marx, B. P. , & Schnurr, P. P. (2013). The PTSD Checklist for DSM‐5 (PCL‐5) . https://www.ptsd.va.gov/professional/assessment/adult‐sr/ptsd‐checklist.asp

[jts23126-bib-0025] Winkielman, P. , Knäuper, B. , & Schwarz, N. (1998). Looking back at anger: Reference periods change the interpretation of emotion frequency questions. Journal of Personality and Social Psychology, 75(3), 719–728. 10.1037//0022-3514.75.3.719 9781408

[jts23126-bib-0026] Winslow, B. D. , Kwasinski, R. , Hullfish, J. , Ruble, M. , Lynch, A. , Rogers, T. , Nofziger, D. , Brim, W. , & Woodworth, C. (2022). Automated stress detection using mobile application and wearable sensors improves symptoms of mental health disorders in military personnel. Frontiers in Digital Health, 4, Article 919626. 10.3389/fdgth.2022.919626 PMC9445306 36082233

