# Supplementary material for: A mixed‐methods investigation of a digital mental health tool to manage posttrauma anger
Source: J Trauma Stress. 2025 Jan 26;38(2):296–304. doi: 10.1002/jts.23126 (PMC11967293; doi:10.1002/jts.23126)
Supplement: Supplementary file 1 — Supporting Material [file JTS-38-296-s001.docx]

Supplementary material

Participant Interview Guide

1. Can you tell me about some factors that might increase your anger, that we haven't captured in this study? You might want to consider emotions, bodily sensations, thoughts, actions, location, social factors in your answer.
2. Can you tell me about some factors that might reduce your anger in the moment? You might want to consider emotions, bodily sensations, thoughts, actions, location, social factors in your answer.
3. Can you tell me about your experience of using the EMA app?
4. Can you tell me about your experience of using the wearable?
5. When you were participating in the study, did anything change about how you managed your anger? What specifically?
6. How did you find the timing and frequency of the daily surveys?
7. Can you tell me about the kinds of things that led to you miss a survey prompt?
8. Can you tell me if any part of the study disrupted or altered your daily life?
9. What did you like about the study?
10. What did you dislike about the study?
11. Is there anything else that made the study difficult or unpleasant to participate in?
12. Can you think of any ways in which the study could be improved?
13. Summarise the reasons overall why data (surveys and wearable) was missing.
